# Periodic Surface Structuring of Copper with Spherical and Cylindrical Lenses

**DOI:** 10.3390/nano13061005

**Published:** 2023-03-10

**Authors:** Meilin Hu, Jijil JJ Nivas, Martina D’Andrea, Mohammadhassan Valadan, Rosalba Fittipaldi, Mariateresa Lettieri, Antonio Vecchione, Carlo Altucci, Salvatore Amoruso

**Affiliations:** 1 Dipartimento di Fisica “Ettore Pancini”, Università degli Studi di Napoli Federico II, Complesso Universitario di Monte S. Angelo, Via Cintia, I-80126 Napoli, Italy; 2 Dipartimento di Scienze Biomediche Avanzate, Università degli Studi di Napoli Federico II, Via Pansini 5, I-80131 Napoli, Italy; 3 CNR-SPIN SuPerconducting and Other INnovative Materials and Devices Institute, UOS Salerno, Via Giovanni Paolo II 132, I-84084 Fisciano, Italy

**Keywords:** laser-induced periodic surface structures, large area processing with circular and elliptical laser beams, femtosecond laser surface structuring with cylindrical lens focusing

## Abstract

The use of a cylindrical lens in femtosecond laser surface structuring is receiving attention to improve the processing efficiency. Here, we investigate the structures produced on a copper target, in air, by exploiting both spherical and cylindrical lenses for beam focusing, aiming at elucidating similarities and differences of the two approaches. The morphological features of the surface structures generated by ≈180 fs laser pulses at 1030 nm over areas of 8 × 8 mm^2^ were analyzed. For the spherical lens, micron-sized parallel channels are formed on the target surface, which is covered by subwavelength ripples and nanoparticles. Instead, the cylindrical lens leads to a surface decorated with ripples and nanoparticles with a negligible presence of micro-channels. Moreover, the morphological features achieved by focusing ≈180 fs laser pulses at 515 nm with the cylindrical lens and varying the scanning parameters were also studied. The experimental results evidence a direct effect of the hatch distance used in the scanning process on the target surface that contains dark and bright bands corresponding to regions where the rippled surface contains a richer decoration or a negligible redeposition of nanoparticles. Our findings can be of interest in large area surface structuring for the selection of the more appropriate focusing configuration according to the final application of the structured surface.

## 1. Introduction

Nano- and micro-scale modifications of solid surfaces by irradiation with laser pulses can result in morphological features that impart interesting functional properties (e.g., optical, chemical, and biological) to material surfaces [1,2,3,4,5,6]. Femtosecond (fs) laser micromachining of solid surfaces allows for the direct creation of periodic features with individual sizes varying from below the laser wavelength up to millimeters, thus offering a promising non-contact method to fabricate functional surfaces. In this context, fs laser structuring continues to attract attention thanks to its applicability to almost any type of material, for its wide technological potential in various fields (e.g., photonics, optoelectronics, micro- and nano-fluidics, biomedicine, and so forth), and to explore the merits of novel laser sources and approaches [7,8,9,10,11,12,13,14,15,16]. In this context, the fundamental process is represented by the formation of self-organized laser-induced periodic surface structures (LIPSS), whose mechanisms are still actively discussed and under investigation [4,17,18,19,20,21]. Most of the studies concern the formation of surface structures characterized by subwavelength spatial period, Λ, indicated as ripples, which are classified into low spatial frequency LIPSS (LFSL), for λ/2 < Λ < λ, and high spatial frequency LIPSS (HSFL), for Λ < λ/2, λ being the laser wavelength. The scalability of the LIPSS period on the laser wavelength allows engineering the surface morphology over a wide range of length scales, thanks to availability of ultrashort laser sources emitting from UV to mid-infrared by means of harmonic generation and optical parametric amplification (OPA). For example, LSFL and HSFL with periods of about 1.6 μm and 0.9 μm were generated on Ge by 3 μm laser pulses obtained by a Ti:Sa-based OPA laser system [22]. More recently, ripples formation on fused silica was addressed by using ≈ 200 fs infrared laser pulses tunable in the (1–3) μm range [23].

An interesting aspect of ripples resides in the possibility of extending their formation to large portions of the target surface by suitably moving the laser beam or the sample [7,24,25], which is particularly relevant for industrial demands of laser processing over a large area. In this respect, diverse strategies can be considered. For example, Le Harzic et al. generated HSFL on Si by exploiting very low energy, 170 fs pulses delivered from a Ti:Sa laser oscillator at 80 MHz repetition rate, tightly focusing the laser beam at a spot size of few μm and scanning it at a mm/s speed [25]. Instead, Gnilitskyi et al. proposed high-speed beam scanning using pulses at sub-MHz pulse repetition rate and pulse fluences well above the ablation threshold of the target material (i.e., in the ablation regime), achieving a typical linear speed of the laser spot on the sample of about 1 m/s by coupling a galvanometric and a motorized stage and producing regular LSFL on silicon [10] and metallic targets [9]. Another interesting approach consists of working at a repetition rate in the 1–10 kHz range and exploiting laser beam focusing with a cylindrical lens, thus scanning a beam elongated in one direction over the target surface to enhance the processing efficiency. For example, Zhang et al. used 1 kHz laser pulses provided by a Ti:Sa system to generate LIPSS on Cu and Ni for wetting applications at scanning speeds of 2–4 mm/s, increasing the processing efficiency by about a factor of ten when using a cylindrical lens with respect to a spherical one [26]. More recently, Chen et al. used a similar approach for the elaboration of LIPSS on a glass coated with an Indium Tin Oxide film, improving the processing efficiency by more than 2 orders of magnitude with respect to that achieved with a spherical lens in the same experimental conditions [27].

Here we report on the morphological features of the structured surface, ensuing beam focusing with spherical or cylindrical lenses, aiming at addressing similarities and differences between the two approaches. Copper was selected as a target material by considering its relevance and wide industrial use in electronic components and devices, thanks to its high electrical and thermal conductivities. Moreover, laser-structured copper with high hydrophobic surfaces is considered of interest, e.g., for compact heat exchangers, water resistant electronics, drag reduction, and micro-electro-mechanical systems (MEMS). More recently, it has also been investigated in the frame of secondary electron yield mitigation for possible applications in high-energy particle accelerators [28]. Further applications of copper nanostructures have been proposed for biosensing and surface enhanced Raman spectroscopy [29,30], in which Cu-based materials can offer a cheaper alternative to noble metal nanostructures, as well as in antimicrobial, biomedical, and agro-food applications [31,32]. LIPSS have also been recently exploited for secondary electron yield (SEY) reduction of metallic surfaces, a process applicable to particle accelerators and space components. In particular, recent studies addressed the possible role of fs-LIPSS on a copper surface with subwavelength-sized features on secondary electron yield SEY [33,34].

The laser processing of copper surface with a Gaussian beam focused with a spherical lens, in air, results in a surface covered by fine ripples, micron-sized channels, or trenches and nanoparticles. Instead, for a cylindrical lens, the surface is covered mainly with LIPSS decorated by nanoparticles and a negligible presence of micro-trenches. Moreover, the scanning parameters greatly influence the optical and morphological properties of the structured surface. The processed area contains dark and bright bands corresponding to areas where the generated LIPSS contain rich decoration and negligible redeposition of nanoparticles, as ascertained from Scanning Electron Microscope (SEM) images, depending on the overlap between scanned lines.

## 2. Experimental Methods

Large area surface processing of Cu samples was carried out using a chirped pulse amplification Yb:KGW laser system (Pharos, Light Conversion) capable of delivering linearly polarized pulses with a duration of ≈180 fs at the fundamental wavelength λ ≈ 1030 nm with a repetition rate variable from single pulse to 200 kHz. Both a spherical and a cylindrical lens were used to focus the laser beam on the copper target surface with the aim of evidencing the similarities and differences of the two processing approaches. The nominal focal length of the spherical and cylindrical lenses were 200 mm and 150 mm, respectively. Prior to the laser-structuring experiments, the laser beam spot sizes on the target surface were measured by analyzing the variation of the area modified by the laser beam irradiation as a function of the laser energy, *E*, following the methods proposed by Liu [35,36,37], which is also supported by theoretical modelling for the case of fs laser irradiation [38]. In the case of the spherical lens, the fluence spatial distribution is described by $F\left(r\right)=F_{p}exp(-2r^{2}/w_{0}^{2})$, where the peak fluence is $F_{p}=\left(2E\right)/\left(\pi w_{0}^{2}\right)$. The estimated beam radius of the circular spot produced by focusing the 1030 nm beam with the spherical lens was estimated to be $w_{0}$ ≈ 77 µm. In the case of the cylindrical lens, the spatial distribution of the beam fluence on the target surface can be described by a Gaussian function with two different spot sizes, $w_{0x}$ and $w_{0y}$, along the *x* and *y* directions, respectively: $F\left(x,y\right)=F_{p}exp\left[-2\left(x^{2}/w_{0x}^{2}+y^{2}/w_{0y}^{2}\right)\right]$, where the peak fluence value is $F_{p}=\left(2E\right)/\left(\pi \;w_{0x}\;w_{0y}\right)$. The values of the spot sizes $w_{0x}$ and $w_{0y}$ were obtained by analyzing the variation of the elliptical area modified by the laser beam irradiation as a function of the laser energy, *E*, and applying the Liu method to the sizes along the *x* and *y* directions. The estimated values of the beam spot sizes at 1030 nm were $w_{0x}$ ≈ 42 μm and $w_{0y}$ ≈ 2.1 mm. In experiments with the cylindrical lens, laser pulses at λ ≈ 515 nm generated by second harmonic generator equipped with a BBO crystal were also exploited, and the beam spot sizes along the *x* and *y* directions were estimated to be $w_{0x}$ ≈ 25 µm and $w_{0y}$ ≈ 2.2 mm, respectively, following the changes in the modified area as a function of the laser energy, *E*. In all cases, the beam spatial distribution on the target surface is well-described by the Gaussian profile reported above.

The copper target was mounted on a computer-controlled XY-translation stage (PP-20, Micronix, Fountain Valley, CA, USA) synchronized with the laser system. During the laser surface structuring experiments, the Cu sample was translated at a velocity $u_{s}$ along the *x*-direction. For the structuring with the cylindrical lens, the direction of sample movement was along the lower elliptical axis of the laser beam spot. For both lenses, a large area surface treatment of 8 × 8 mm^2^ was achieved by moving the sample in a boustrophedonic way, scanning along the *x*-direction at a velocity $u_{s}$ and then moving by a fixed step along the *y*-direction, with a given hatch distance *Δy*. A given amount of beam overlap was assured in both directions by proper selection of the values of the repetition rate *f_p_*, the scanning speed $u_{s}$, and the hatch distance *Δy*. In all cases, the laser beam polarization was oriented along the *x*-direction. After the laser treatment, the surface morphology was analyzed by optical and scanning electron microscopies. The optical micrographs were taken using a Nikon Eclipse LV100ND optical microscope (Nikon Corp., Tokyo, Japan) equipped with a Nikon DS-Fi3 camera. The NIS-Elements Br software was used for image acquisition and data processing. The scanning electron microscope (SEM) images were registered by using a field emission scanning electron microscope (Zeiss ∑IGMA, Oberkochen, Germany) through secondary electrons (SE) detection at 20 kV.

## 3. Results and Discussion

Here we discuss the influence of the irradiation conditions on the features of the processed surface. We first examined the effect of the different focusing conditions on the efficiency of the structuring procedure, i.e., the time it takes to process the same sample surface area under two different conditions. The beam spot achieved with the spherical lens is of the order of 100 μm, while the cylindrical lens allows for forming a beam elongated in one direction over a few mm, while keeping the beam size along the orthogonal direction at values comparable to that of the spherical lens, i.e., several tens of μm. For the sake of estimation, one can consider the same value of the beam spot size along the scanning direction for the two lenses and a fixed scanning velocity; therefore, more than one order of magnitude increase of the surface processing rate can be expected. For instance, at a scan velocity of 1 mm/s, for the processing at 1030 nm, the estimated processing rate is $2w_{0}u_{s}$ ≈ 0.16 mm^2^/s for the spherical lens and $2w_{0y}u_{s}$ ≈ 4.2 mm^2^/s for the cylindrical one, respectively; hence, the corresponding processing time for an area of 8 × 8 mm^2^ reduces from about 20 min to less than 1 min. However, the different beam characteristics and related hatch distances to cover the same processed area also affect the morphological features of the processed surface, which can be of importance for the final applications. In the following, we will address such an aspect by discussing some of the experimental results obtained with the spherical and cylindrical lenses.

**Figure 1:**
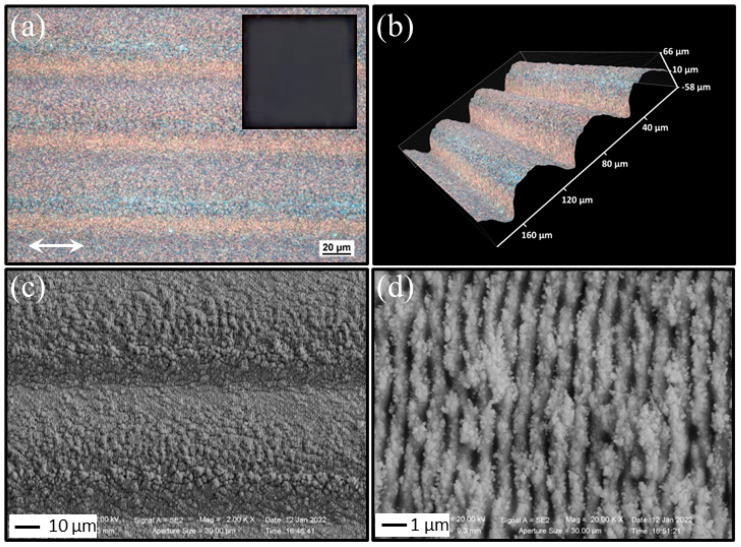
Exemplificative optical microscope and SEM images of the processed copper surface. The images refer to a sample processed with the spherical lens at 1030 nm at a repetition rate *f_p_* = 20 kHz and a scanning speed $u_{s}$ = 1 mm/s. The pulse energy is *E* ≈ 70 μJ and the corresponding peak fluence is *F_p_* ≈ 0.75 J/cm^2^. The hatch distance is *Δy* = 55 µm. Panel (**a**) shows a microscope image. Here, the scanning direction is set along the polarization direction of the beam identified by the double headed arrow. A photograph of the processed sample of size 8 mm × 8 mm is given in the inset of panel (**a**). Panel (**b**) reports a 3D image of the processed area evidencing the formation of channels along the scan direction. Panels (**c**,**d**) report SEM images of the processed surface addressing the generation of subwavelength ripples and the surface decoration with nanoparticles.

Here we discuss the features of the sample surface, which is processed using a spherical lens at 1030 nm. Figure 1 reports, as an example, the characteristic morphology of the copper sample surface after processing, in air, at a repetition rate of 20 kHz and a pulse energy *E* ≈ 70 µJ; the corresponding peak fluence is $F_{p}\approx$0.75 J/cm^2^. The scanning velocity is $u_{s}$= 1 mm/s, and the hatch distance is *Δy* = 55 µm. The inset of panel (a) displays a photograph, in dark grey colors, of the sample surface under visible light evidencing the 8×8 mm^2^ processed surface area, whereas Figure 1a reports a zoomed view obtained by the optical microscope. Here, different zones in the form of periodic parallel lines along the *y*-direction, separated by the selected hatch distance *Δy*, can be clearly recognized. A three-dimensional (3D) view of the structured area is reported in Figure 1b, which clearly shows the presence of channels in the structured area; this image was acquired by exploiting an option of the optical microscope that allows a 3D image reconstruction through the acquisition of several 2D images at different distances from the objective. The depth of these channels varies as a function of the pulse fluence, repetition rate, and scanning speed. Figure 1c reports an SEM image displaying the finer surface morphology of the structured surface from which the formation of the channels along the scanning direction, as well as the generation of well-developed LIPSS, can also be observed. The LIPSS can be better visualized in Figure 1d, which reports a SEM image registered at higher resolution, which evidences a surface densely decorated with subwavelength ripples covered by nanoparticles. Hence, the processing with the spherical lens results in a surface characterized by a triple-scale hierarchical structure, which includes: (i) a pattern of very shallow parallel micro-trenches (or channels); (ii) a finer texture of subwavelength LIPSS (period Λ = 816 ± 45 µm), whose orientation depends on the laser beam polarization direction; (iii) random decoration of nanoparticles ensuing the surface ablation and back-deposition on the target surface for processing at atmospheric pressure [39,40].

The features illustrated above are typical of the bidirectional scanning of a circular Gaussian beam over a large area, where finer characteristics such as the shallow parallel micro-trenches depth and period of the LIPSS features depend on the scanning configuration and laser pulse parameters [41]. In the conditions used for the elaboration of the sample reported in Figure 1, the overlap between the laser beam spots is larger than 99.9% along the scanning *x*-direction and ≈ 65% along the *y*-direction, which should result in a rather uniform distribution of the total fluence dose distribution. Nevertheless, the boustrophedonic scanning procedure needed to cover the large area progressively modifies the surface imprinting on it the track covered by the intense laser beam, and eventually gives rise to the formation of the channels decorated with LIPSS and nanoparticles observed in Figure 1.

**Figure 2:**
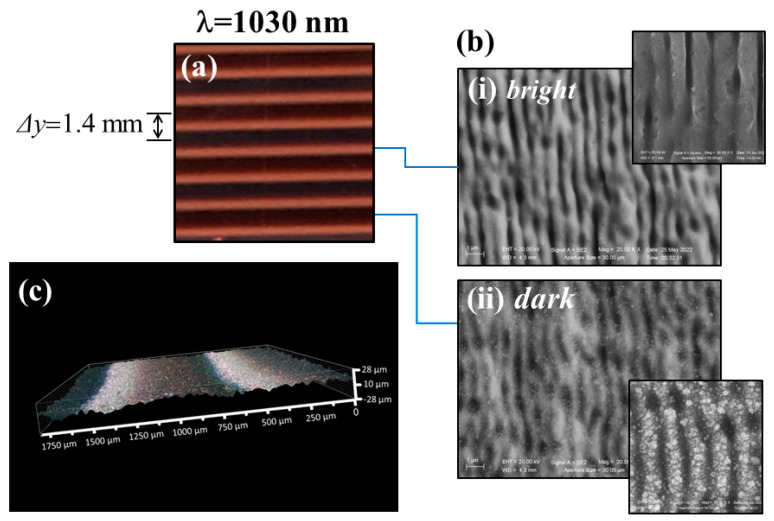
Exemplificative optical and SEM images of the copper sample processed with the cylindrical lens focusing at λ = 1030 nm, with a repetition rate *f_p_* = 5 kHz and scanning speed $u_{s}$ = 1 mm/s, a pulse energy *E* ≈ 10 μJ (*F_p_* ≈ 0.37 J/cm^2^), and hatch distance *Δy* = 1.4 mm. Panel (**a**) displays a photograph of the sample surface evidencing the formation of bright and dark stripes. Panel (**b**) reports SEM images of the corresponding bright and dark bands, respectively. The insets of the SEM images show zoomed views in the two bright and dark regions illustrating the finer morphology of the ripples. Panel (**c**) shows the 3D image of the processed surface.

We turn now to the processing with the cylindrical lens illustrating the typical feature of samples elaborated by using the same laser beam at 1030 nm. As an example, Figure 2a reports a photograph of the structured area after irradiating the sample at a repetition rate of 5 kHz and a pulse energy *E* ≈ 10 µJ; the corresponding peak fluence is $F_{p}$ ≈ 0.37 J/cm^2^. The scanning velocity is $u_{s}$ = 1 mm/s, and the hatch distance is *Δy* = 1.4 mm. As can be observed from the image of Figure 2a, the surface of the structured copper sample shows several parallel stripes of different colors separated by a distance corresponding to the hatch distance *Δy*.

Panel (b) of Figure 2 reports SEM images registered in the bright and dark bands. The SEM images collected in the dark bands appear to be grainier in comparison to those of the bright bands, which evidence better defined ripples. All the sample surface is covered by subwavelength ripples oriented in direction perpendicular to the laser beam polarization. The spatial period of the ripples in the dark band is Λ =(840 ± 60) nm, whereas that measured in the bright zone results in being slightly shorter, i.e., Λ = (780 ± 50) nm. This is consistent with the general observation that the ripple spatial period decreases with the effective number of pulses hitting the target surface, since bright stripes correspond to regions with the highest number of pulses [2,12]. This is because the bright region is formed by the less intense part of the beam, and this region is where the scan lines overlap. Compared to the dark region that corresponds to the intense part of the beam, the overlapped regions between the scan lines experience almost twice as many pulses. In Figure 2, the insets (i) and (ii) of panel (b) report high-resolution SEM images of the two regions. As can be observed, the ripples generated in the dark stripes are covered by a thick layer of nanoparticles, whereas those in the bright bands are almost free of nanoparticle decoration. Therefore, the darker color can possibly be ascribed to a larger coverage of nanoparticles; instead, the bright stripes may result from the negligible number of nanoparticles and the predominance of subwavelength ripples characterized by a comparatively smoother surface morphology. The negligible coverage by nanoparticles in the bright regions can be likely due to a washing out effect of the back-deposited nanoparticles in this region, ensuing ablation or melting processes by the overlapping part of the beam during line scanning. Finally, Figure 2c reports a 3D image of the processed surface with cylindrical lens acquired to visualize a few scan lines. This further ensures that the formation of channels or micro-trenches is minimal or almost negligible in the case of the cylindrical lens.

**Figure 3:**
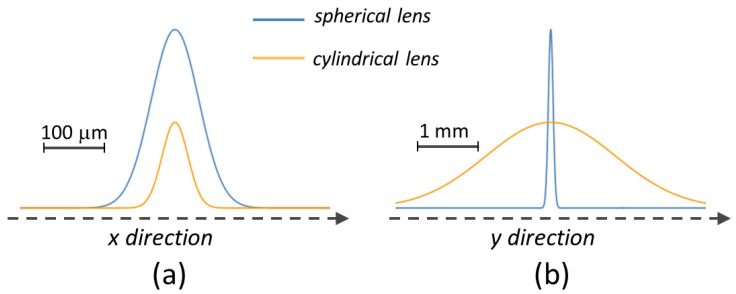
A sketch of the spatial profiles of the laser beams produced by the spherical and cylindrical lenses on the target surface along the (**a**) *x*-direction and (**b**) *y*-direction. The scale bars in the two panels evidence the different spatial scales along the scanning *x*-direction and the stepping y-direction, respectively. The proportion of the peak fluence corresponds to the factor of ≈2 between values of the peak fluence for the spherical (Figure 1) and cylindrical (Figure 2) lenses.

It is worth observing that the conditions of Figure 2 correspond to an overlap between the elliptical laser beam spots of ≈ 99.8% along the scanning *x*-direction and ≈67%, along the *y*-direction, values rather similar to those of the previous case with the spherical lens. However, the spatial distributions of the laser fluence along the *y*-direction are rather different for the two cases, whereas those along the *x*-direction are somewhat comparable. This is shown in Figure 3, which schematically reports the beam spatial profiles on the target surface along the *x* and *y* directions as produced by the spherical and cylindrical lenses, respectively, for the experimental parameters of the laser beams discussed above. The fluence profiles along the *x*-direction have a similar extension, but the spatial distribution of the laser pulse energy is highly concentrated in the *y*-direction for the spherical lens and is spread over the surface for the cylindrical one. The highly peaked fluence distribution associated with the spherical lens is responsible for the formation of channels along the scanning track in this configuration. Such an effect is largely mitigated by the spreading of the fluence occurring for the cylindrical lens, eventually resulting only in the formation of stripes characterized by a slightly different ripples period and nanoparticle coverage. The sizable difference in the density of redeposited particle in the diverse stripes is likely associated to a change in the expansion and redeposition dynamics of the ablated plume in the two cases. In the case of a Gaussian beam focused by a spherical lens, the pressure experienced by the expanding plume will be ideally uniform in all directions around the axis of the incident beam. However, this is not true in the case of cylindrical lenses. In such irradiation cases, particles will be more redeposited along the longer part of the elliptical spot crated by the cylindrical lens. Preliminary experiments carried out in static irradiation conditions confirm a different spatial distribution of the nanoparticles around the shallow crater formed on the sample surface [42]. In particular, a sequence of *N* = 200 laser pulses was used to irradiate the sample, in air, and in conditions similar to those used for large area processing. For the spherical lens, a circular shallow crater was formed, which was surrounded by an annular area largely covered by nanoparticles extending over a spatial width of the order of ≈20% of the crater radius, in agreement with the results reported by Semaltianos et al. [39]. Instead, for the cylindrical lens, the elliptical crater is decorated on the two longer sides with a strip whose width depends on the laser fluence and reduces to a very thin region at lower fluences as those exploited above. Hence, this suggests that the rather large coverage of nanoparticles observed in the case of the spherical lens results from the nanoparticulate debris remaining on both the upper and lower sides of the track and behind the scanning laser spot, thus also redecorating the inner part of the channels progressively formed on the surface. Instead, in the case of the cylindrical lens, the different spatial distribution of the nanoparticulate debris can result in a more effective melting and ablation of the nanoparticles back-deposited in previous laser shots, due to the large overlap between the laser pulses along the *x*-direction and the more limited extension of the nanoparticles stripes. These effects are more pronounced in the central region of the elliptical beam with higher fluence leaving nanoparticles namely on the wings of the scanning elliptical beam along the major axis, as was observed above. Such dynamics will eventually lead to the formation of the bright and dark bands observed for the large area structuring with the elliptical beam. The nanoparticles expansion and redeposition dynamics in the various experimental conditions and its dependence on the experimental parameters was investigated only in a few cases [39,40] and deserves further investigations to clearly address the influence of the beam spot shape on its features.

Interestingly, fs laser surface structuring in air allows for both the formation of well-defined surface nano-gratings and the production of nanoparticles and nanoparticles assembled structures, depending on the specific experimental configurations. Both these surface morphologies can be of interest to develop low-cost plasmonic nanomaterials. Several applications of laser-structured surfaces have been already addressed in relation to their intriguing optical, wetting, mechanical, and biological-active properties [1,2,3,4,5]. However, they might express their potential in other fields like, for example, those pursuing the elaboration of nano-engineered plasmonic surfaces for SERS and metal-enhanced fluorescence [30,31,32,43,44,45,46].

**Figure 4:**
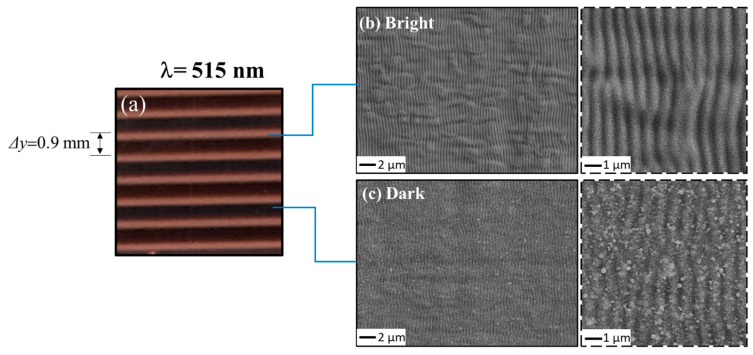
Exemplificative optical and SEM images of the copper sample processed with the cylindrical lens focusing at λ = 515 nm, with a repetition rate *f_p_* = 5 kHz and scanning speed $u_{s}$ = 0.5 mm/s, a pulse energy E ≈ 4.5 μJ (*F_p_* ≈ 0.4 J/cm^2^) and hatch distance *Δy* = 0.9 mm. Panel (**a**) displays a photograph of the sample surface evidencing the formation of bright and dark stripes. Panels (**b**,**c**) report SEM images of the corresponding bright and dark bands, respectively. The insets of the SEM images show zoomed views in the two bright and dark regions, illustrating the finer morphology of the ripples.

For the sake of further investigation, we also carried out experiments on large area processing with the cylindrical lens using the beam at a wavelength of 515 nm. Additionally, in this case, samples with an 8 × 8 mm^2^ area were processed. Here, the repetition rate was set at *f_p_* = 5 kHz and the scanning speed at $u_{s}$ = 0.5 mm/s. The pulse energy used was *E* ≈ 4.0 μJ for a corresponding peak fluence *F_p_* ≈ 0.4 J/cm^2^. As an example, Figure 4a displays a photo of the sample obtained for a hatch distance *Δy* = 0.9 mm. As can be observed, the general features of the processed area are like those already observed at 1030 nm, with the formation of parallel bright and dark stripes. Figure 4b,c and the corresponding insets illustrate the finer morphology of the sample surface in the two regions confirming also in this case the formation of well-defined ripples with a smoother surface in the bright regions and a nanoparticles coverage in the dark ones. The ripples periods are Λ =(390 ± 25) µm and Λ =(440 ± 28) µm for the bright and dark stripes, respectively. The measured values of the ripple periods are coherent with those observed in the case of 1030 nm irradiation, i.e., Λ is lower in the bright region, corresponding to the dark area where the local fluence is also higher.

**Figure 5:**
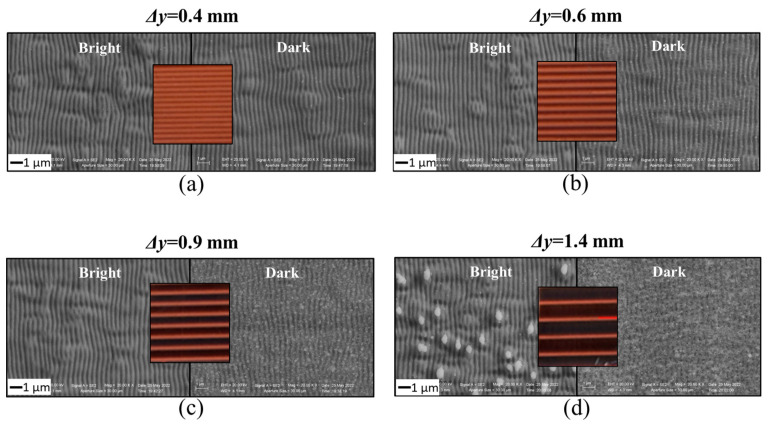
Photos and SEM images of the copper surface processed at 515 nm with the cylindrical lens for different values of the hatch distance *Dy*: (**a**) 0.4 mm, (**b**) 0.6 mm, (**c**) 0.9 mm, (**d**) 1.4 mm. The repetition rate is *f_p_* = 5 kHz and the pulse energy is *E* ≈ 4 μJ; the corresponding peak fluence is *F_p_* ≈ 0.42 J/cm^2^. The photos showcase the variation of the spatial disposition of the bright and dark bands as a function of the hatch distance *Δy*. The SEM images display the typical surface morphological features of the bright and dark stripes in various cases.

We also analyzed the effect of the hatch distance variation at this wavelength, as shown in Figure 5, which reports photos and SEM images of the structured copper surface for four different values of *Δy* from 0.4 mm to 1.4 mm while keeping the other parameters, as in Figure 4. The central photo in each panel is surrounded by the SEM images for the bright and dark bands, respectively. As can be observed from the photographs, the width of the dark band is maximum when *Δy* is the highest and progressively reduces as *Δy* decreases; the overlap between adjacent lines in the *y*-direction increases from ≈ 68% at *Δy* = 1.4 mm up to 91% at *Δy* = 0.4 mm, while the overlap along the scanning *x*-direction is ≈ 99%. For *Δy* = 0.4 mm, only very thin dark bands are observed, as can be seen in the Figure 5a. As for the finer morphological features, the SEM images illustrate, in a fairly clear way, a dependence of the surface morphology on the hatch distance *Δy*: the surface roughness is different in bright and dark areas. Like the cases discussed above in Figure 2 and Figure 4, independently of the specific value of *Δy*, the bright regions contain smooth ripples, whereas the dark zones are richly decorated with nanoparticles. Furthermore, as *Δy* reduces, a gradual change in the amount of nanoparticle decorating the dark band is observed. In more specific terms, the presence of nanoparticles found in the dark band considerably reduced as *Δy* changes from 1.4 mm to 0.4 mm. When it comes to *Δy* = 0.4 mm case, there is no considerable change in the surface morphology between bright and dark regions as the last becomes progressively less covered by nanoparticles. Hence, it is likely that the change of the overlap fraction can be a rather good parameter to control the nanoparticle coverage in the case where smoother ripples should be need, but at the expenses of the processing time which passes from ≈90 s for *Δy* = 1.4 mm to ≈300 s for *Δy* = 0.4 mm for an 8 × 8 mm^2^ processed area. The formation of observably different regions with a sizable difference in the density of nanoparticles likely arises from the change in the focal spot and associated expansion and redeposition dynamics of the ablated plume, an aspect that deserves further investigations in the near future. However, the experimental findings reported above clearly evidence the possibilities offered by cylindrical lens focusing in obtaining smoother surfaces with a limited formation of channels, which are inherent to the exploitation of a spherical lens. Nonetheless, the specific experimental configuration should be selected by keeping in mind the final application of the processed surface, as the functional response can significantly depend on the surface morphology, like that addressed, for example, for the dependence of the wetting properties of laser-treated copper on the depth of the channels produced by the spherical lens beam focused on in our previous study [41], or for copper and nickel structured with a beam focused by a cylindrical lens, as reported by Zhang et al. [26].

## 4. Conclusions

We reported an experimental investigation on fs laser surface structuring of a copper target in dynamic irradiation conditions. The surface was processed over areas of 8×8 mm^2^ by exploiting both spherical and cylindrical lenses for beam focusing, with the aim of elucidating similarities and differences of the two approaches. In fact, the former is the most used method but needs fast scanning methodology to be highly efficiency due to the typical size of the circular beam on the target. The latter makes use of an elliptical beam with a size of few mm in the longer direction that can help in enhancing the efficiency even at lower scanning velocity. Of course, the different beam features and scanning procedures will induce changes in the final features of the structured surface.

The spherical lens focusing results in a surface covered by fine ripples, micron-sized channels, and nanoparticles. These features are typical of the bidirectional scanning of a circularly symmetric, intense Gaussian beam over a large area. Instead, cylindrical lens focusing produces a surface covered mainly with ripples decorated by nanoparticles and a negligible presence of micro-trenches. Interestingly, the surface of the samples processed with the cylindrical lens focusing display dark and bright bands corresponding to regions where the ripples contain rich decoration or negligible redeposition of nanoparticles, respectively. Our experimental results evidence a direct effect of the hatch distance used in the bidirectional scanning procedure and the possibility to limit the micro-channels by appropriate selection of the experimental parameters. Our experimental findings can be of interest in large area surfaces, structuring for the selection of the more appropriate focusing configuration according to the final application of the structured surface. Among possible applications of interest one can quote, for example, the mitigation of secondary electron yield for copper screen for particles accelerator colliders [33,34], the development of Cu-based substrates plasmonic sensors for biosensing and antibacterial applications [29,30,31,32], the elaboration of surfaces with peculiar wetting features [41,47], and so forth. Our results also address how spatially shaped beams can add further flexibility to fs laser processing, even by a simple change of the focusing lens, besides the more complex approaches based on complex beam shaping and spatially variant polarization illustrated in previous publications [3,6,11,12,48].

## Data Availability

Not applicable.
